# Supplementary material for: Pharmacokinetics of Sodium and Calcium Salts of (6S)-5-Methyltetrahydrofolic Acid Compared to Folic Acid and Indirect Comparison of the Two Salts
Source: Nutrients. 2020 Nov 25;12(12):3623. doi: 10.3390/nu12123623 (PMC7760477; doi:10.3390/nu12123623)
Supplement: Supplementary file 1 [file nutrients-12-03623-s001.zip › nutrients-948749-supplementary-proof/nutrients-948749-proof-supplementary figure.pptx]

## Slide 1
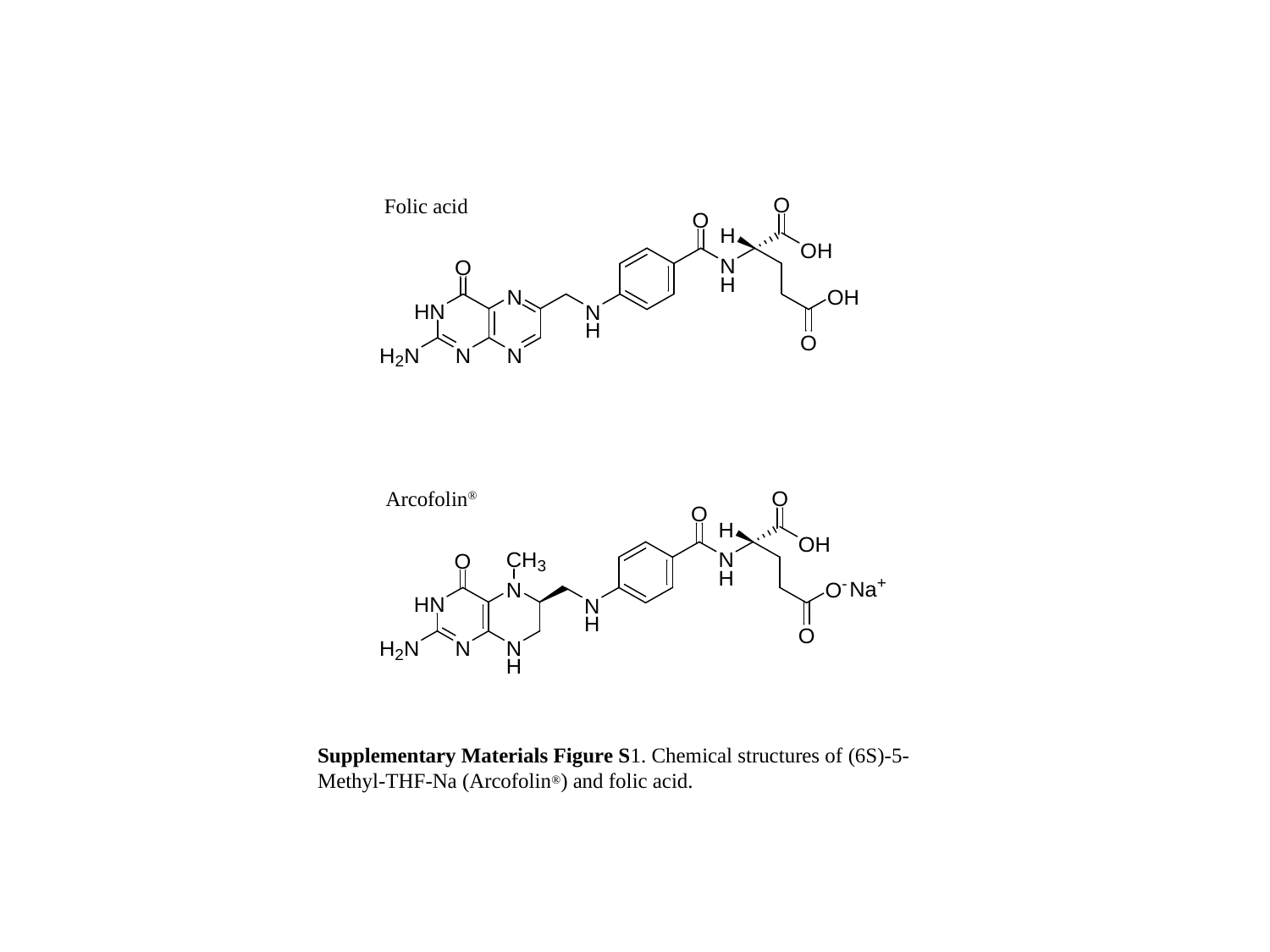

Folic acid
Arcofolin®
Supplementary Materials Figure S1. Chemical structures of (6S)-5-Methyl-THF-Na (Arcofolin®) and folic acid.

## Slide 2
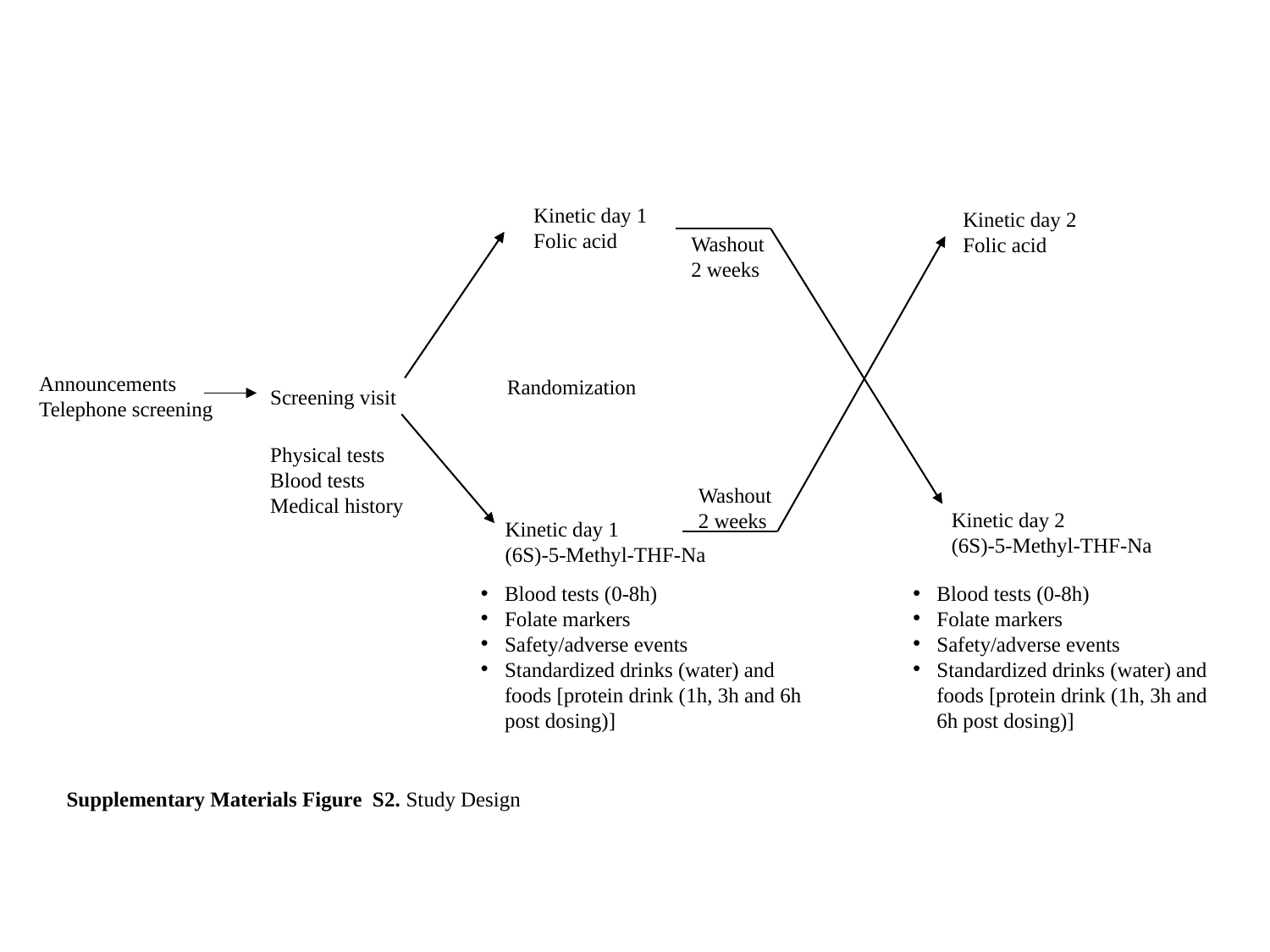

Kinetic day 1
Folic acid
Kinetic day 2
Folic acid
Washout
2 weeks
Announcements
Telephone screening
Randomization
Screening visit
Physical tests
Blood tests
Medical history
Kinetic day 2
(6S)-5-Methyl-THF-Na
Kinetic day 1
(6S)-5-Methyl-THF-Na
Blood tests (0-8h)
Folate markers
Safety/adverse events
Standardized drinks (water) and foods [protein drink (1h, 3h and 6h post dosing)]
Blood tests (0-8h)
Folate markers
Safety/adverse events
Standardized drinks (water) and foods [protein drink (1h, 3h and 6h post dosing)]
Supplementary Materials Figure S2. Study Design
Washout
2 weeks

## Slide 3
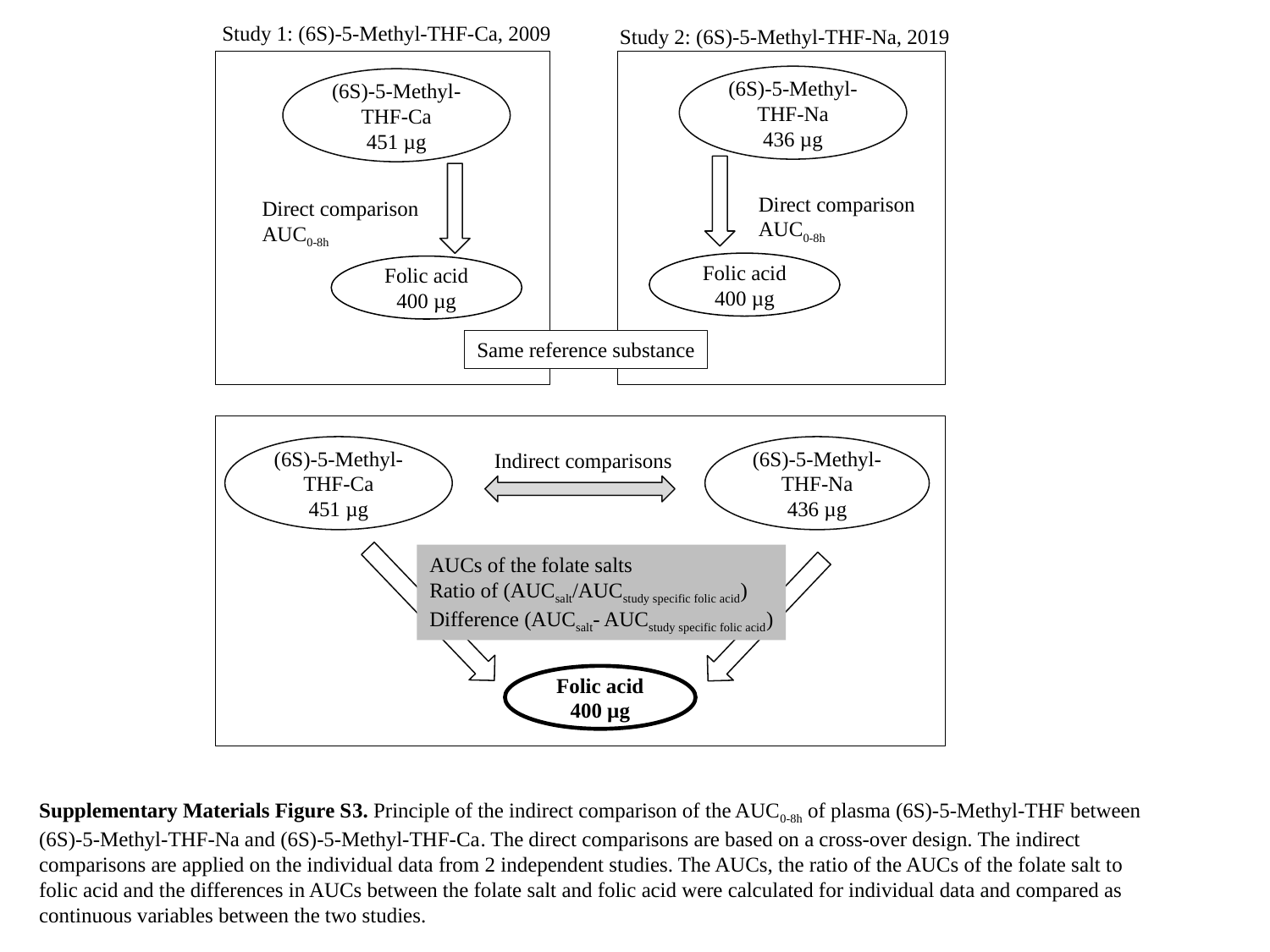

Study 1: (6S)-5-Methyl-THF-Ca, 2009
Study 2: (6S)-5-Methyl-THF-Na, 2019
(6S)-5-Methyl-THF-Na
436 µg
(6S)-5-Methyl-THF-Ca
451 µg
Direct comparison
AUC0-8h
Direct comparison
AUC0-8h
Folic acid
400 µg
Folic acid
400 µg
Same reference substance
(6S)-5-Methyl-THF-Ca
451 µg
(6S)-5-Methyl-THF-Na
436 µg
Indirect comparisons
AUCs of the folate salts
Ratio of (AUCsalt/AUCstudy specific folic acid)
Difference (AUCsalt- AUCstudy specific folic acid)
Folic acid
400 µg
Supplementary Materials Figure S3. Principle of the indirect comparison of the AUC0-8h of plasma (6S)-5-Methyl-THF between (6S)-5-Methyl-THF-Na and (6S)-5-Methyl-THF-Ca. The direct comparisons are based on a cross-over design. The indirect comparisons are applied on the individual data from 2 independent studies. The AUCs, the ratio of the AUCs of the folate salt to folic acid and the differences in AUCs between the folate salt and folic acid were calculated for individual data and compared as continuous variables between the two studies.

## Slide 4
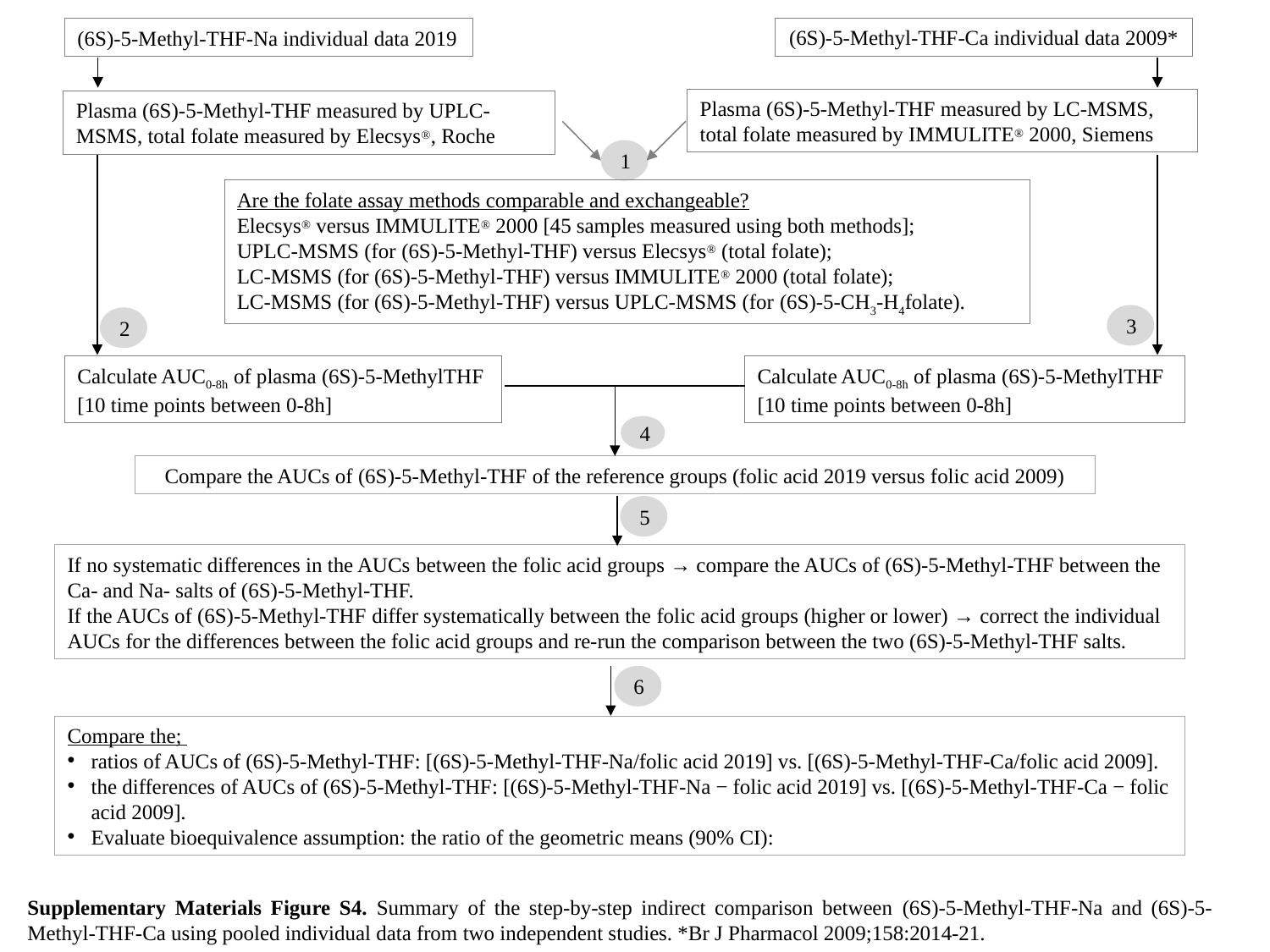

(6S)-5-Methyl-THF-Ca individual data 2009*
(6S)-5-Methyl-THF-Na individual data 2019
Plasma (6S)-5-Methyl-THF measured by LC-MSMS,
total folate measured by IMMULITE® 2000, Siemens
Plasma (6S)-5-Methyl-THF measured by UPLC-MSMS, total folate measured by Elecsys®, Roche
1
Are the folate assay methods comparable and exchangeable?
Elecsys® versus IMMULITE® 2000 [45 samples measured using both methods];
UPLC-MSMS (for (6S)-5-Methyl-THF) versus Elecsys® (total folate);
LC-MSMS (for (6S)-5-Methyl-THF) versus IMMULITE® 2000 (total folate);
LC-MSMS (for (6S)-5-Methyl-THF) versus UPLC-MSMS (for (6S)-5-CH3-H4folate).
3
2
Calculate AUC0-8h of plasma (6S)-5-MethylTHF
[10 time points between 0-8h]
Calculate AUC0-8h of plasma (6S)-5-MethylTHF
[10 time points between 0-8h]
4
Compare the AUCs of (6S)-5-Methyl-THF of the reference groups (folic acid 2019 versus folic acid 2009)
5
If no systematic differences in the AUCs between the folic acid groups → compare the AUCs of (6S)-5-Methyl-THF between the Ca- and Na- salts of (6S)-5-Methyl-THF.
If the AUCs of (6S)-5-Methyl-THF differ systematically between the folic acid groups (higher or lower) → correct the individual AUCs for the differences between the folic acid groups and re-run the comparison between the two (6S)-5-Methyl-THF salts.
6
Compare the;
ratios of AUCs of (6S)-5-Methyl-THF: [(6S)-5-Methyl-THF-Na/folic acid 2019] vs. [(6S)-5-Methyl-THF-Ca/folic acid 2009].
the differences of AUCs of (6S)-5-Methyl-THF: [(6S)-5-Methyl-THF-Na − folic acid 2019] vs. [(6S)-5-Methyl-THF-Ca − folic acid 2009].
Evaluate bioequivalence assumption: the ratio of the geometric means (90% CI):
Supplementary Materials Figure S4. Summary of the step-by-step indirect comparison between (6S)-5-Methyl-THF-Na and (6S)-5-Methyl-THF-Ca using pooled individual data from two independent studies. *Br J Pharmacol 2009;158:2014-21.

## Slide 5
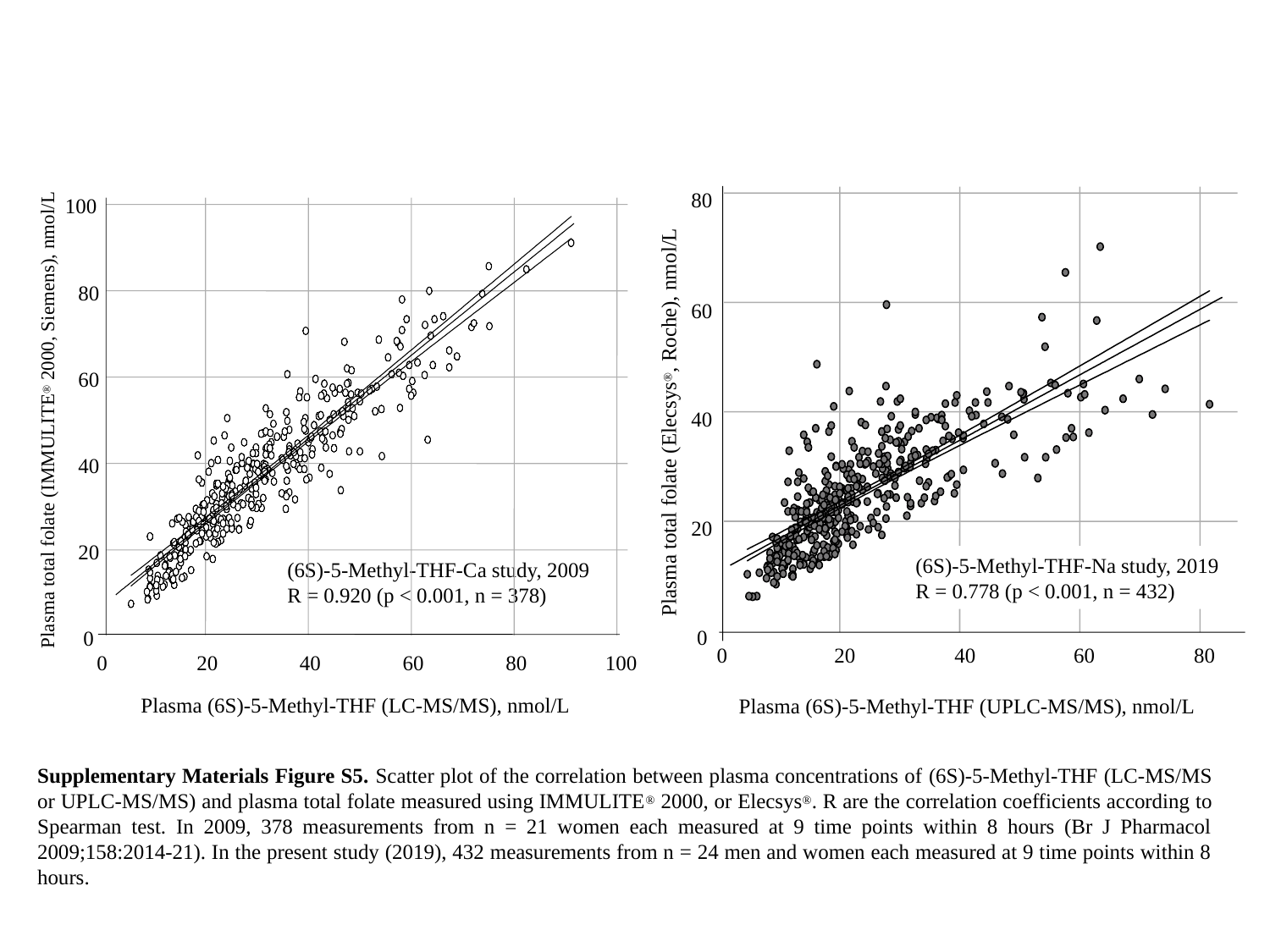

80
60
40
Plasma total folate (Elecsys®, Roche), nmol/L
20
0
0
20
40
60
80
Plasma (6S)-5-Methyl-THF (UPLC-MS/MS), nmol/L
100
80
60
Plasma total folate (IMMULITE® 2000, Siemens), nmol/L
40
20
(6S)-5-Methyl-THF-Ca study, 2009
R = 0.920 (p < 0.001, n = 378)
0
0
20
40
60
80
100
Plasma (6S)-5-Methyl-THF (LC-MS/MS), nmol/L
(6S)-5-Methyl-THF-Na study, 2019
R = 0.778 (p < 0.001, n = 432)
Supplementary Materials Figure S5. Scatter plot of the correlation between plasma concentrations of (6S)-5-Methyl-THF (LC-MS/MS or UPLC-MS/MS) and plasma total folate measured using IMMULITE® 2000, or Elecsys®. R are the correlation coefficients according to Spearman test. In 2009, 378 measurements from n = 21 women each measured at 9 time points within 8 hours (Br J Pharmacol 2009;158:2014-21). In the present study (2019), 432 measurements from n = 24 men and women each measured at 9 time points within 8 hours.

## Slide 6
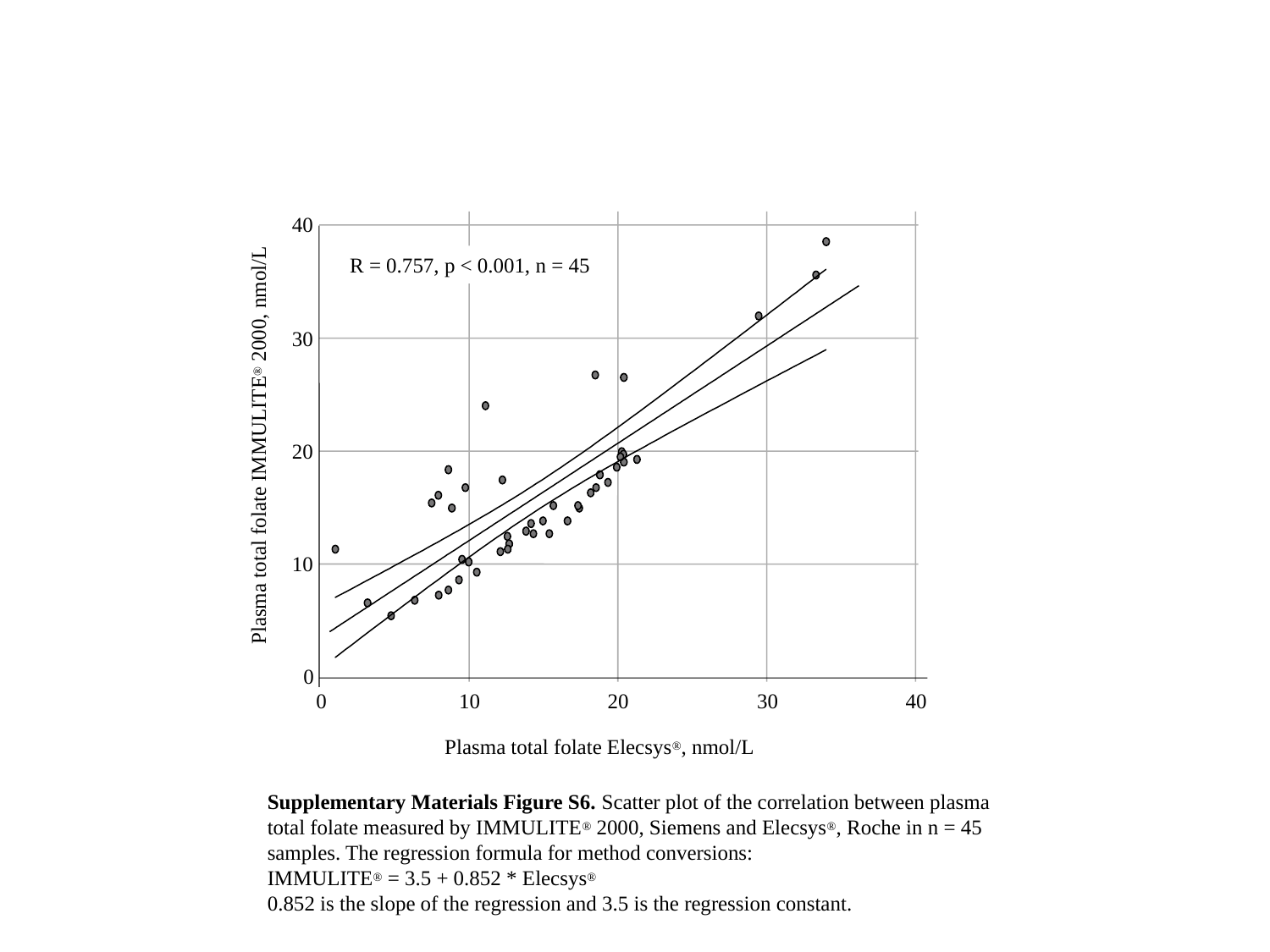

40
R = 0.757, p < 0.001, n = 45
30
Plasma total folate IMMULITE® 2000, nmol/L
20
10
0
0
10
20
30
40
Plasma total folate Elecsys®, nmol/L
Supplementary Materials Figure S6. Scatter plot of the correlation between plasma total folate measured by IMMULITE® 2000, Siemens and Elecsys®, Roche in n = 45 samples. The regression formula for method conversions:
IMMULITE® = 3.5 + 0.852 * Elecsys®
0.852 is the slope of the regression and 3.5 is the regression constant.

## Slide 7
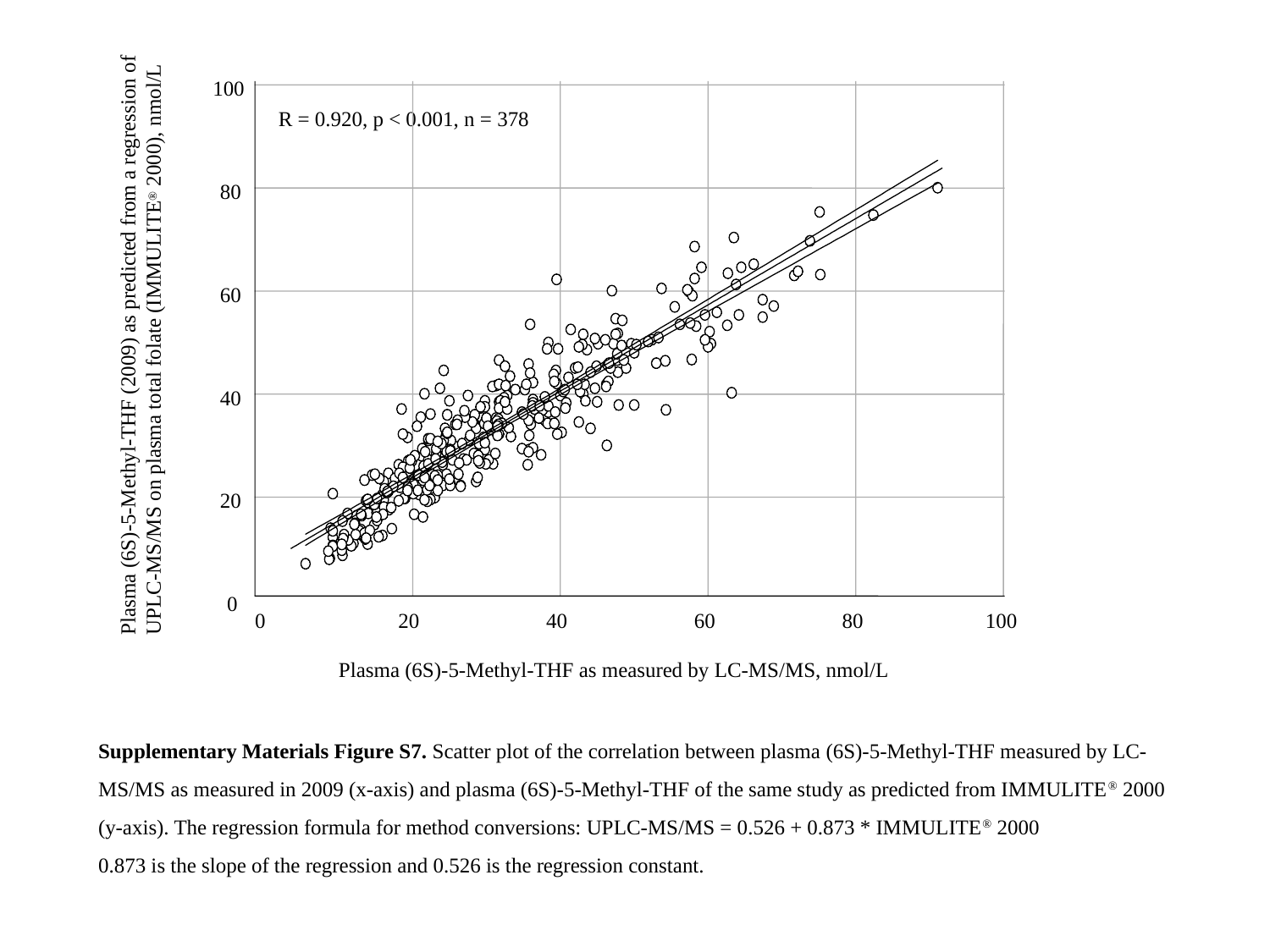

100
R = 0.920, p < 0.001, n = 378
80
60
40
20
0
0
20
40
60
80
100
Plasma (6S)-5-Methyl-THF as measured by LC-MS/MS, nmol/L
Plasma (6S)-5-Methyl-THF (2009) as predicted from a regression of UPLC-MS/MS on plasma total folate (IMMULITE® 2000), nmol/L
Supplementary Materials Figure S7. Scatter plot of the correlation between plasma (6S)-5-Methyl-THF measured by LC-MS/MS as measured in 2009 (x-axis) and plasma (6S)-5-Methyl-THF of the same study as predicted from IMMULITE® 2000 (y-axis). The regression formula for method conversions: UPLC-MS/MS = 0.526 + 0.873 * IMMULITE® 2000
0.873 is the slope of the regression and 0.526 is the regression constant.

## Slide 8
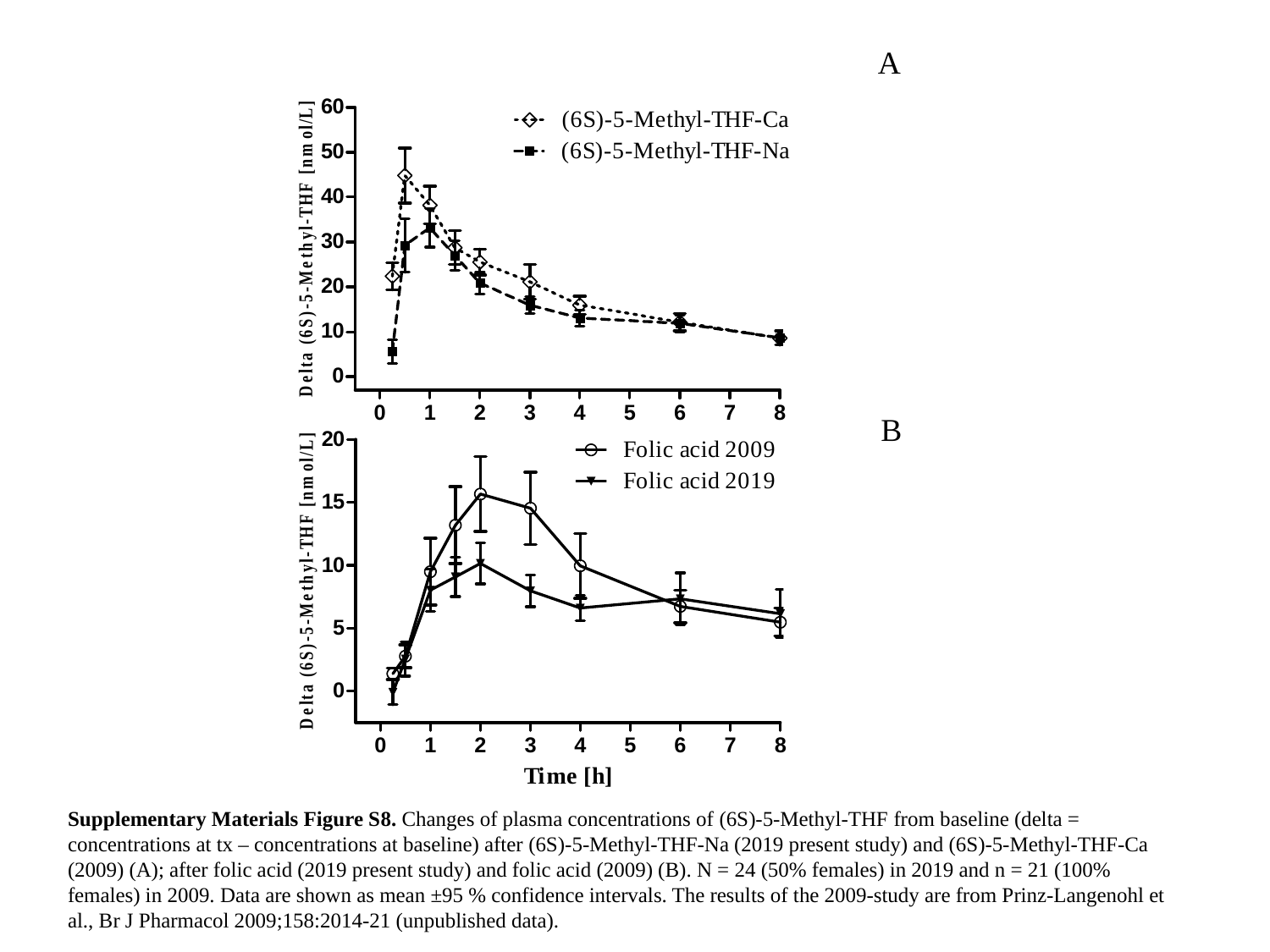

A
B
Supplementary Materials Figure S8. Changes of plasma concentrations of (6S)-5-Methyl-THF from baseline (delta = concentrations at tx – concentrations at baseline) after (6S)-5-Methyl-THF-Na (2019 present study) and (6S)-5-Methyl-THF-Ca (2009) (A); after folic acid (2019 present study) and folic acid (2009) (B). N = 24 (50% females) in 2019 and n = 21 (100% females) in 2009. Data are shown as mean ±95 % confidence intervals. The results of the 2009-study are from Prinz-Langenohl et al., Br J Pharmacol 2009;158:2014-21 (unpublished data).

## Slide 9
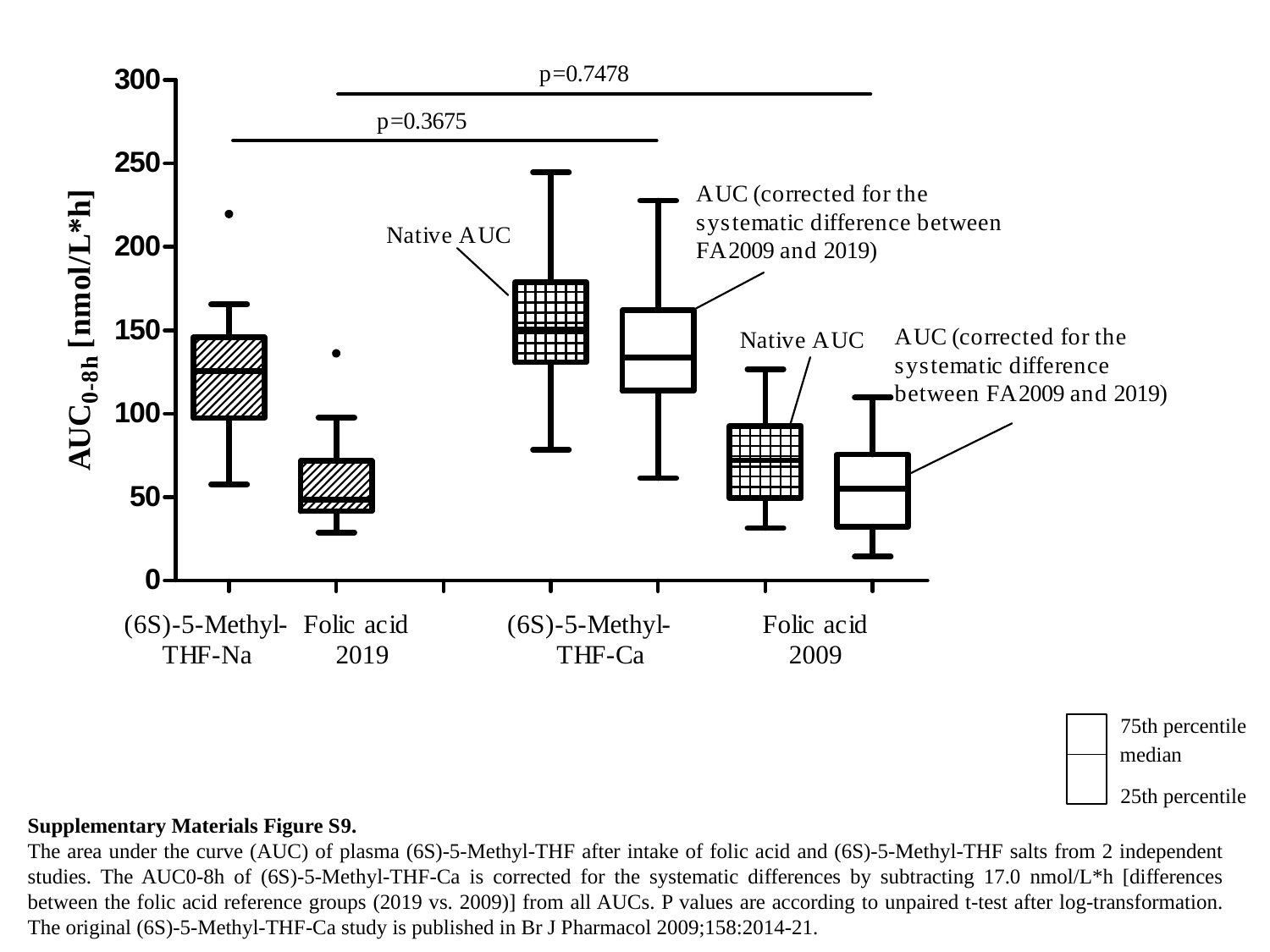

75th percentile
median
25th percentile
Supplementary Materials Figure S9.
The area under the curve (AUC) of plasma (6S)-5-Methyl-THF after intake of folic acid and (6S)-5-Methyl-THF salts from 2 independent studies. The AUC0-8h of (6S)-5-Methyl-THF-Ca is corrected for the systematic differences by subtracting 17.0 nmol/L*h [differences between the folic acid reference groups (2019 vs. 2009)] from all AUCs. P values are according to unpaired t-test after log-transformation. The original (6S)-5-Methyl-THF-Ca study is published in Br J Pharmacol 2009;158:2014-21.
