# Supplementary material for: Pharmacokinetics of Sodium and Calcium Salts of (6S)-5-Methyltetrahydrofolic Acid Compared to Folic Acid and Indirect Comparison of the Two Salts
Source: Nutrients. 2020 Nov 25;12(12):3623. doi: 10.3390/nu12123623 (PMC7760477; doi:10.3390/nu12123623)
Supplement: Supplementary file 1 [file nutrients-12-03623-s001.zip › nutrients-948749-supplementary-proof/nutrients-948749-proof-supplementary table.docx]

| **Supplementary Materials Table S1.** Summary of the designs of the pharmacokinetic studies that were used for indirect comparisons of the AUCs for (6S)-5-Methyl-THF-Na (present study) and (6S)-5-Methyl-THF-Ca [Br J Pharmacol 2009;158:2014-21]. | | |
| --- | --- | --- |
| Key design points | (6S)-5-Methyl-THF-Na [Arcofolin^®^, present] | (6S)-5-Methyl-THF-Ca [Metafolin^®^, 2009] |
| Study location | Stuttgart, Germany | Bonn, Germany |
| Participants | 12 women, 12 men | 24 women |
| Test substance, dose | Arcofolin^®^: 436 µg (6S)-5-Methyl-THF-Na | Metafolin^®^: 451 µg (6S)-5-Methyl-THF-Ca |
| Comparator | Folic acid, 400 µg | Folic acid, 400 µg |
| Design | randomized, double-blind, cross over | randomized, double-blind, cross over |
| Blood collection time points | 0,0.25,0.5,1, 2, 3, 4, 6, 8h | 0,0.5,1, 1.5, 2, 3, 4, 6, 8h |
| Plasma (6S)-5-Methyl-THF assay | UPLCMSMS (Homburg, Germany) | LCMSMS (Amsterdam, The Netherlands) |
| Plasma total folate immunoassay | Elecsys^®^ (Cobas, Roche), Electrochemiluminescence | IMMULITE^®^2000, Siemens, a competitive chemiluminescence |
| Main exclusion criteria | Low and high plasma folate <7 and > 45 nM; RBC folate < 405 and > 952 nM; smoking; anemia; B12 <148 pM; Homocysteine ≥ 15μM; taking supplements during the last 3 months | Supplement use, smoking, anaemia, plasma folate < 6.8 nM, RBC-folate <317 nM and plasma B12 <110 pM. |
| Main inclusion criteria | Healthy adults | Healthy women, MTHFR677TT (n=16)+CC(n=8) |
| Diet during intervention | Standardized folate free protein drink | Standardized folate free protein drink |
| Outcome | Primary: AUC_0-8h_ of plasma (6S)-5-Methyl-THF Secondary: AUC_0-8h_ of plasma total folate | Primary: AUC_0-8h_ of plasma total folate;  Secondary: AUC_0-8h_ of plasma (6S)-5-Methyl-THF |

| **Supplementary Materials Table S2**. Concentrations of plasma (6S)-5-Methyl-THF over 8 hours and the corresponding AUC_0-8h_ in the present study [(6S)-5-Methyl-THF-Na versus folic acid] and in the 2009 study [(6S)-5-Methyl-THF-Ca versus folic acid (1)]. | | | | |  |
| --- | --- | --- | --- | --- | --- |
| Plasma (6S)-5-Methyl-THF, nmol/L | **Present study** | | **2009** | |  |
|  | (6S)-5-Methyl-THF-Na | **Reference**  **Folic acid** | (6S)-5-Methyl-THF-Ca | **Reference**  **Folic acid** |  |
| t0 | 18.0 (18.0) | **16.7 (7.6)** | 14.9 (7.1) | **17.1 (7.7)** |  |
| t0.25h | 36.9 (14.7)^b^ | **20.0 (10.1)^b^** | 37.3 (9.7) | **18.5 (7.6)** |  |
| t0.5h | 55.7 (19.6) | **23.4 (15.7)** | 59.7 (15.1) | **19.9 (7.6)** |  |
| t1h | 50.0 (15.0) | **28.3 (13.4)** | 53.1 (12.1) | **26.6 (11.1)** |  |
| t1.5h | 41.3 (12.9) | **30.8 (12.1)** | 43.6 (11.6)^a^ | **30.3 (12.0)^a^** |  |
| t2h | 38.6 (11.3) | **33.0 (11.8)** | 40.4 (10.5) | **32.8 (11.8)** |  |
| t3h | 34.4 (11.6) | **31.1 (11.1)** | 36.0 (11.4) | **31.7 (11.3)** |  |
| t4h | 29.3 (9.7) | **26.3 (10.7)** | 30.8 (9.3) | **27.0 (11.0)** |  |
| t6h | 25.6 (9.4) | **22.7 (8.9)** | 27.1 (9.0) | **23.9 (8.8)** |  |
| t8h | 22.1 (8.3) | **21.7 (7.5)** | 23.5 (8.0) | **22.6 (7.5)** |  |
| AUC_0-8h_, nmol/L * h | 126.0 (33.6) | **56.0 (25.3)^c^** | 152.9 (36.5) | **73.1 (26.5)** |  |
| Results are shown as mean (SD). ^a^ (6S)-5-Methyl-THF concentrations at t1.5h are calculated from individual values as the mean of levels at t1h and t2h in 2019. ^b^ (6S)-5-Methyl-THF levels at t0.25h are calculated from individual values as the mean of levels at t0 and t0.5h in 2009.  ^c^ AUC of plasma (6S)-5-Methyl-THF was systematically higher in 2009 than in 2019 by17.0 nmol/L*h. | | | | |  |

| **Supplementary Materials Table S3**. Comparisons of the AUC_0-8h_ in the present study [(6S)-5-Methyl-THF-Na vs. folic acid] and in the 2009 study [(6S)-5-Methyl-THF-Ca vs. folic acid (1)]. | | | |
| --- | --- | --- | --- |
|  | Folic acid 2019 | Folic acid 2009 |  |
| AUC_0-8h_, nmol/L*h | 56.0 (25.3) | 73.1 (26.5) | Difference (95%CI) = 17.0 (1.43, 32.6 ); p = 0.0203^b^ |
|  | (6S)-5-Methyl-THF-Na | (6S)-5-Methyl-THF-Ca |  |
| AUC_0-8h_, nmol/L*h | 126.0 (33.6) | 152.9 (36.5) |  |
|  | AUCs of (6S)-5-Methyl-THF-Na/ AUC folic acid 2019 | AUCs (6S)-5-Methyl-THF-Ca/  folic acid 2009 |  |
| AUC Ratio | 2.52 (1.02) | 2.29 (0.76) | p = 0.4392 (Mann-Whitney U test) |
|  | AUC folic acid 2019 – AUC (6S)-5-Methyl-THF-Na | AUC folic acid 2009 – AUC (6S)-5-Methyl-THF-Ca |  |
| AUC Difference | -69.9 (29.0) | -79.9 (34.3) | p = 0.2986^c^ |
|  | (6S)-5-Methyl-THF-Na (native) | (6S)-5-Methyl-THF-Ca (corrected)^a^ |  |
| AUC_0-8h_, nmol/L*h | 126.0 (33.6) | 135.9 (36.5)^a^ | p = 0.3675^b^ |
|  |  |  | Ratio of the geometric means (90% CI) = 0.93 (0.80, 1.07) |
| Results are shown as mean (SD).  ^a^ the AUC_0-8h_ of (6S)-5-Methyl-THF-Ca is corrected for the systematic differences by subtracting 17.0 nmol/L*h [differences between the folic acid reference groups (2019 vs. 2009)] from all AUCs.  ^b^ P values are according to unpaired t-test applied on the log transformed data.  ^c^ P values are according to unpaired t-test | | | |

**Supplemental text: Additional statistical analyses for comparisons of the AUC of (6S)-5-Methyl-THF-Na and (6S)-5-Methyl-THF-Ca**

**Dealing with different analytical methods in the independent studies**

The plasma concentrations of (6S)-5-Methyl-THF were measured using LC-MS/MS method in 2009 and UPLC-MS/MS method in 2019. Both assay methods use isotope-labelled-internal standards and can selectively detect (6S)-5-Methyl-THF. Plasma total folate concentrations were measured using immunological assays; in 2009 (IMMULITE^®^ 2000, Siemens) and in 2019 (Elecsys^®^, Roche). Plasma concentrations of (6S)-5-Methyl-THF and those of total folate showed strong correlations within the studies (**Supplementary Materials Figure S5**). This shows a high within-study agreement of (6S)-5-Methyl-THF and total folate assay methods and thereby supports the within study validity of the AUCs. To ensure the external validity of the folate immunological assays, we next compared IMMULITE^®^ 2000 and Elecsys^®^ in 45 plasma samples collected in the (6S)-5-Methyl-THF-Na study (2019). The concentrations measured by IMMULITE^®^ 2000 and Elecsys^®^ were strongly correlated (correlation coefficient R = 0.757, p<0.001, n = 45) **Supplementary Materials Figure S6**.

Using different methods in the 2 pharmacokinetic studies is not likely to introduce a bias in calculating the AUCs because the calculation of the AUCs takes the baseline concentrations as the null for computing the increase above baseline over the 8h. Additional regression analyses were performed to convert plasma folate measured by Elecsys^®^ to IMMULITE^®^ 2000 to ensure interchangeability of the assays when comparing the AUCs between the studies. **Supplementary Materials Figure S7** shows the correlation between the concentrations of plasma (6S)-5-Methyl-THF as measured by LC-MS/MS (x-axis) vs. the predicted UPLC-MS/MS concentrations from the same study as estimated from the regression of UPLC-MS/MS from the 2019 study (dependent variable) and IMMULITE^®^ 2000 (independent variable) (y-axis). UPLC-MS/MS = 0.526 + 0.873 * IMMULITE^®^ 2000

0.873 is the slope of the regression and 0.526 is the regression constant.
